# Structural and functional rescue of cones carrying the most common cone opsin C203R missense mutation

**DOI:** 10.1172/jci.insight.172834

**Published:** 2024-01-23

**Authors:** Emily R. Sechrest, Xiaojie Ma, Marion E. Cahill, Robert J. Barbera, Yixiao Wang, Wen-Tao Deng

**Affiliations:** 1 Department of Ophthalmology and Visual Sciences, West Virginia University, Morgantown, West Virginia, USA.; 2 Department of Ophthalmology, University of Florida, Gainesville, Florida, USA.; 3 Department of Biology and; 4 Department of Biochemistry and Molecular Medicine, West Virginia University, Morgantown, West Virginia, USA.

**Keywords:** Ophthalmology, Gene therapy, Genetic diseases, Protein misfolding

## Abstract

An arginine to cysteine substitution at amino acid position 203 (C203R) is the most common missense mutation in human cone opsin. Linked to color blindness and blue cone monochromacy (BCM), C203 is involved in a crucial disulfide bond required for proper folding. It has previously been postulated that expression of mutant C203R cone opsin exerts a toxic effect on cone photoreceptors, similar to some well-characterized missense mutations in rhodopsin that lead to protein misfolding. In this study, we generated and characterized a BCM mouse model carrying the equivalent C203R mutation (*Opn1mw^C198R^* *Opn1sw*^–/–^) to investigate the disease mechanism and develop a gene therapy approach for this disorder. Untreated *Opn1mw^C198R^* *Opn1sw*^–/–^ cones phenocopied affected cones in human patients with the equivalent mutation, exhibiting shortened or absent cone outer segments and loss of function. We determined that gene augmentation targeting cones specifically yielded robust rescue of cone function and structure when *Opn1mw^C198R^* *Opn1sw^–/–^* mice were treated at early ages. Importantly, treated cones displayed elaborated outer segments and replenished expression of crucial cone phototransduction proteins. Interestingly, we were unable to detect OPN1MW^C198R^ mutant opsin at any age. We believe this is the first proof-of-concept study exploring the efficacy of gene therapy in BCM associated with a C203R mutation.

## Abstract 2

<p>Gene therapy restores the structure and function of cone photoreceptors carrying the most common C203R missense mutation in cone opsin.</p>

## Introduction

Blue cone monochromacy (BCM) is an X-linked inherited visual disorder characterized by loss of functional L- and M-cone photoreceptors due to mutations in the *OPN1LW/OPN1MW* gene cluster coding for L- (*OPN1LW*) and M-opsin (*OPN1MW*). L- and M- photoreceptors, which express long-wavelength–sensitive (L-) or medium-wavelength–sensitive (M-) opsin, respectively, are highly concentrated in the fovea, a structure at the center of the retina that is responsible for high-acuity vision (1–7). Patients with BCM typically present with severely reduced visual acuity and impaired color vision but also exhibit infantile nystagmus, photophobia, and often, myopia (8, 9). The genes coding for L- and M-opsin exist in a head-to-tail tandem array located on the X chromosome, with 1 copy of *OPN1LW* followed by 1 or more copies of *OPN1MW* (10). Typically, only the first 2 genes in the array are expressed due to their proximity to the upstream regulatory locus control region (LCR). Due to the high sequence homology between these genes and their proximity to one another, unequal homologous recombination events are common, resulting in gene fusion, deletion, or duplication (11–13). While several mechanisms have been shown to cause BCM, the 2 most common BCM-causing mutations in the *OPN1LW/MW* gene cluster include large deletion mutations in the upstream LCR that lead to abolished expression of both L- and M-opsin, as well as a Cys203Arg (C203R) missense mutation in a single hybrid L-/M-opsin gene and in a downstream M-opsin gene (4, 14–19).

Detailed studies have compared the retinal structure of patients with BCM with these mutations, demonstrating that BCM retinas from both groups exhibit foveal cones with significantly shortened or absent cone outer segments (COSs) and an uncharacteristically thin outer nuclear layer in the foveal region that continues to show increased thinning with aging (20, 21). It was originally hypothesized that patients with a C203R missense mutation may show a more severe phenotype, as this mutation results in the expression of a misfolded protein that is retained in the endoplasmic reticulum (ER), potentially causing ER stress (15, 21, 22). Surprisingly, BCM patients with a C203R mutation demonstrated a slower rate of development of inner segment (IS) and outer segment defects and decades-longer persistence of foveal outer nuclear layer thickness compared with patients with deletion mutations, though at later stages in life, patients had comparable losses of foveal structure (20, 21). Most importantly, while patients with BCM of either genotype do experience cone degeneration, the remaining cones retain an intact IS, which houses the cell’s biosynthesis machinery, suggesting that these cones remain viable targets for gene therapy.

Visual opsin proteins contain 2 highly conserved cysteines that are involved in a disulfide bond that is crucial for proper folding; a c.607 T>C mutation (p.C203R) disrupts this interaction, resulting in translation of a misfolded protein that is presumably retained in the ER. Patients carrying a corresponding mutation in rhodopsin (C187Y) display early and severe autosomal dominant retinitis pigmentosa (adRP) (23). As L-/M-opsin genes are X-linked, affected males express only the mutant copy, while the cones of female carriers will express either a wild-type (WT) or mutant copy due to X-linked inactivation. Along these lines, it is unknown whether the currently identified BCM-associated cone opsin missense mutants possess any dominant-negative properties.

We have shown previously that gene replacement therapy can rescue both function and COS structure in BCM cones with deletion mutations using *Opn1mw^–/–^* and *Opn1mw^–/–^* *Opn1sw^–/–^* (double knockout, DKO) mice (24–28). In the current study, we generated and validated a mouse model of BCM carrying the equivalent human C203R missense mutation (*Opn1mw^C198R^* *Opn1sw^–/–^*). Using this model, we demonstrate that gene augmentation therapy results in elaboration of the COS, replenishment of COS proteins, and restoration of cone function. We believe our study is the first demonstration of successful gene therapy to treat cones carrying the C203R missense mutation in cone opsin and provides proof of concept toward future clinical trials to restore vision in patients with BCM.

## Results

### Characterization of Opn1mw^C198R^ and Opn1mw^C198R^ Opn1sw^–/–^ mice.

**Figure 1:**
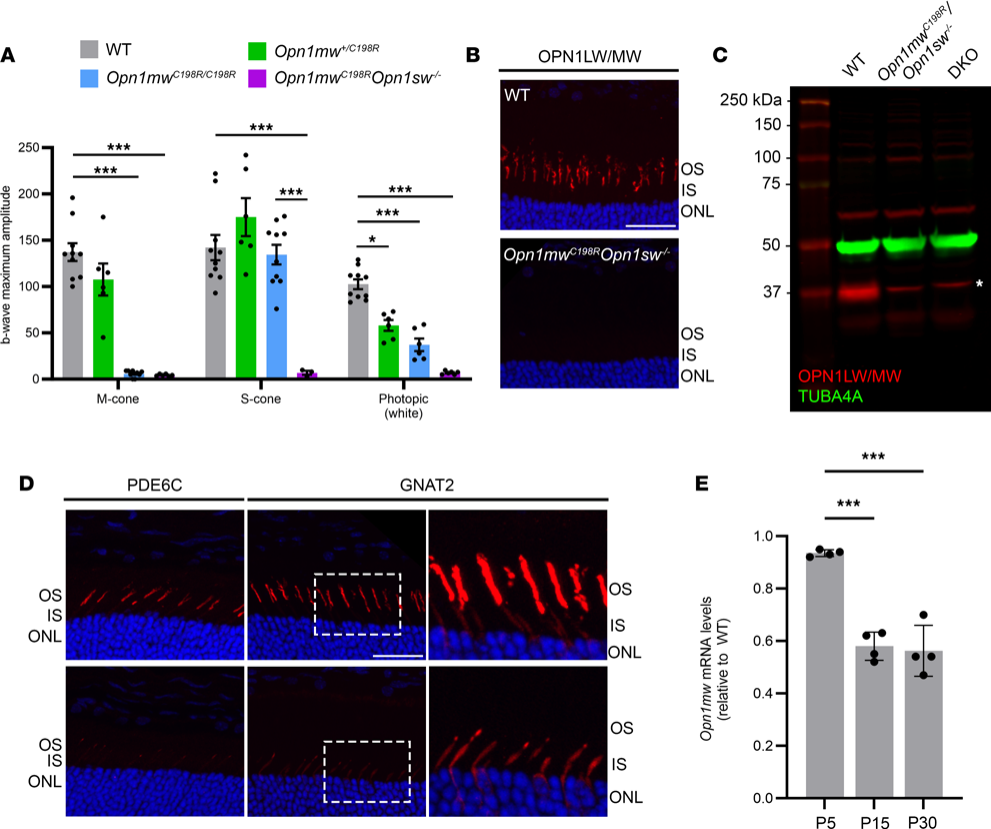
*Opn1mw^C198R^Opn1sw^–/–^* mice lack cone function and demonstrate severely reduced expression of COS-specific proteins. (**A**) ERG b-wave maximum amplitude with medium-wavelength (M-cone, 25 cd●s/m^2^), short-wavelength (S-cone, 2.5 cd●s/m^2^), and white (photopic, 25 cd●s/m^2^) light for WT (gray, *n* = 10), *Opn1mw^+/C198R^* heterozygous female (green; *n* = 6), *Opn1mw^C198R/C198R^* female (blue; *n* = 6), and *Opn1mw^C198R^* *Opn1sw^–/–^* (purple; *n* = 6, *n* = 3 for S-cone only) mice. Data represented as mean ± SEM, 2-way ANOVA (*P ≤ 0.05, ***P < 0.001). ERG was performed on mice at 1 month of age. Statistical significance was noted only for comparisons between WT and other groups or between *Opn1mw^C198R/C198R^* and *Opn1mw^C198R^* *Opn1sw^–/–^* mice. (**B** and **C**) Opn1mw^C198R^ mutant opsin was not detected by IHC and Western blot at P30. (**B**) Representative IHC images of WT and *Opn1mw^C198R^* *Opn1sw^–/–^* cross sections stained with antibody against L-/M-opsin. Scale bar = 20 μm. (**C**) Western blot analysis of retinal lysates of WT (left lane), *Opn1mw^C198R^* *Opn1sw^–/–^* (middle lane), and *Opn1mw^–/–^* *Opn1sw^–/–^* DKO (right lane) showing the endogenous expression level of M-opsin (OPN1MW, red). TUBA4A (green) was used as a loading control. DKO retinal lysate served as a negative control for M-opsin. Asterisk notes a nonspecific band generated by the L-/M-opsin antibody. (**D**) Representative IHC images of WT and *Opn1mw^C198R^* *Opn1sw^–/–^* cross sections labeled with antibodies against PDE6C (left panel) and GNAT2 (middle panel). Oversaturation of GNAT2 staining (right panel) shows GNAT2 staining in WT cross sections is mainly in the COS, with minimal labeling in the IS, whereas *Opn1mw^C198R^* *Opn1sw^–/–^* cones exhibit exclusive staining of GNAT2 to the IS. PDE6C expression was barely detectable in *Opn1mw^C198R^* *Opn1sw^–/–^* eyes. In contrast, PDE6C was expressed and localized exclusively to the COS in WT eyes. Scale bar = 20 μm. Original magnification: the rightmost images in **D** are the 40× images seen in the middle images. (**E**) Real-time quantitative PCR of *Opn1mw^C198R^* mRNA levels in *Opn1mw^C198R^* *Opn1sw^–/–^* retinas at P5, P15, and P30 relative to *Opn1mw* mRNA in age-matched WT controls (*n* = 3). Data represented as mean ± SD, 1-way ANOVA (***P < 0.001). OS, outer segment; ONL, outer nuclear layer; TUBA4A, tubulin alpha-4A.

To establish a new model of BCM caused by the most common missense mutation, C203R, we first characterized retinal function and opsin expression in *Opn1mw^C198R^* knockin mice by electroretinography (ERG) and immunohistochemistry (IHC), respectively. *Opn1mw^C198R^* mice demonstrated undetectable M-cone–mediated (λ = 510 nm) ERG responses but normal S-cone– (λ = 368 nm) and rod-mediated ERG responses. These mice also showed significantly reduced photopic white light–mediated function, likely due to loss of M-cone function (Figure 1A). Retinal cross sections from homozygous female or hemizygous male *Opn1mw^C198R^* mice displayed normal staining and expression of S-opsin to the COS by IHC; however, mutant OPN1MW^C198R^ protein was not detected. Consistently, cone photoreceptor cells positive for M-opsin staining in heterozygous *Opn1mw^+/C198R^* females were about half of those observed in WT controls (Supplemental Figure 1; supplemental material available online with this article; https://doi.org/10.1172/jci.insight.172834DS1).

The majority of mouse cones coexpress M- and S- opsin in an opposing dorsal-ventral gradient with more abundant M-opsin expression in the dorsal retina and S-opsin in the ventral retina (29). In contrast, cone photoreceptors in the human retina express only 1 type of opsin; therefore, affected BCM cones presumably only express C203R-opsin. To generate a mouse model that takes this fact into account and to better model BCM cones at the cellular level, we next crossed *Opn1mw^C198R^* mice with *Opn1sw^–/–^* mice to generate *Opn1mw^C198R^* *Opn1sw^–/–^* mice. This mouse model resulted in the exclusive expression of only OPN1MW^C198R^ mutant opsin in cones, similar to L- and M-cones in patients with BCM. This design provided an optimal model for characterizing the disease mechanism caused by the C203R missense mutation, as well as for assessing the efficacy of gene therapy without interference from endogenous S-opsin. We then characterized retinal function and opsin expression in these mice. Middle-wavelength light–, short-wavelength light–, and white light–mediated ERG analysis showed that *Opn1mw^C198R^* *Opn1sw^–/–^* cones lacked any photopic function (Figure 1A and Supplemental Figure 2, A and B), while rod function was normal (Supplemental Figure 2C). No differences were noted between WT and *Opn1mw^C198R^* *Opn1sw^–/–^* retinas when eyes were evaluated by fundus imaging (Supplemental Figure 3A) or optical coherence tomography (O.C.T.) (Supplemental Figure 3B) at any tested age.

IHC labeling of *Opn1mw^C198R^* *Opn1sw^–/–^* retinal cross sections at postnatal day (P) 30 showed undetectable OPN1MW^C198R^ expression (Figure 1B). Additionally, we were unable to detect OPN1MW^C198R^ protein at P15 in *Opn1mw^C198R^* *Opn1sw^–/–^* cross sections (Supplemental Figure 4). Immunoblot analysis of retinal lysates from WT, *Opn1mw^C198R^* *Opn1sw^–/–^*, and *Opn1mw^–/–^* *Opn1sw^–/–^* (DKO) (27) mice at P30 showed normal M-opsin expression in WT retinas but verified loss of M-opsin expression from both *Opn1mw^C198R^* *Opn1sw^–/–^* and DKO retinas (Figure 1C). To rule out the possibility that our antibody against L-/M-opsin is incapable of recognizing mutant OPN1MW^C198R^, we transfected HEK293T cells with plasmids expressing either mutant OPN1MW^C198R^ opsin or a WT OPN1MW-HA control. These experiments verified that this antibody can detect both WT and mutant opsin by immunoblot analysis (Supplemental Figure 5).

We also utilized antibodies against phosphodiesterase-6 subunit α′ (PDE6C) and cone transducin subunit α (GNAT2) to assess localization of COS-specific proteins in *Opn1mw^C198R^* *Opn1sw^–/–^* cones by IHC. PDE6C expression was barely detectable in *Opn1mw^C198R^* *Opn1sw^–/–^* retinas while age-matched WT sections showed normal expression and COS localization (Figure 1D, left panel). Expression of GNAT2 was also dramatically decreased in *Opn1mw^C198R^* *Opn1sw^–/–^* mice, with minimal staining localized within the cone IS (Figure 1D, middle and right panels). These results indicate that COSs are substantially shortened or missing in *Opn1mw^C198R^* *Opn1sw^–/–^* retinas.

Next, quantitative real-time PCR was used to analyze *Opn1mw* mRNA levels in *Opn1mw^C198R^* *Opn1sw^–/–^* and WT retinas at P5, P15, and P30. We found that *Opn1mw^C198R^* mRNA was normal at P5 but was reduced approximately 42% by P15 and by 44% at P30 compared with WT, suggesting there might be a feedback mechanism to reduce the gene expression of mutant *Opn1mw^C198R^* (Figure 1E). Moreover, mRNA levels of *Pde6c* and *Gnat2* in *Opn1mw^C198R^* *Opn1sw^–/–^* mice remained similar to WT mice at 1 month of age (Supplemental Figure 6). Taken together, these data suggest that while gene expression of *Opn1mw^C198R^* is being regulated at the mRNA level, OPN1MW^C198R^ protein is likely being translated but is subsequently degraded.

### Assessing the rate of cone degeneration in Opn1mw^C198R^ Opn1sw^–/–^ mice.

**Figure 2:**
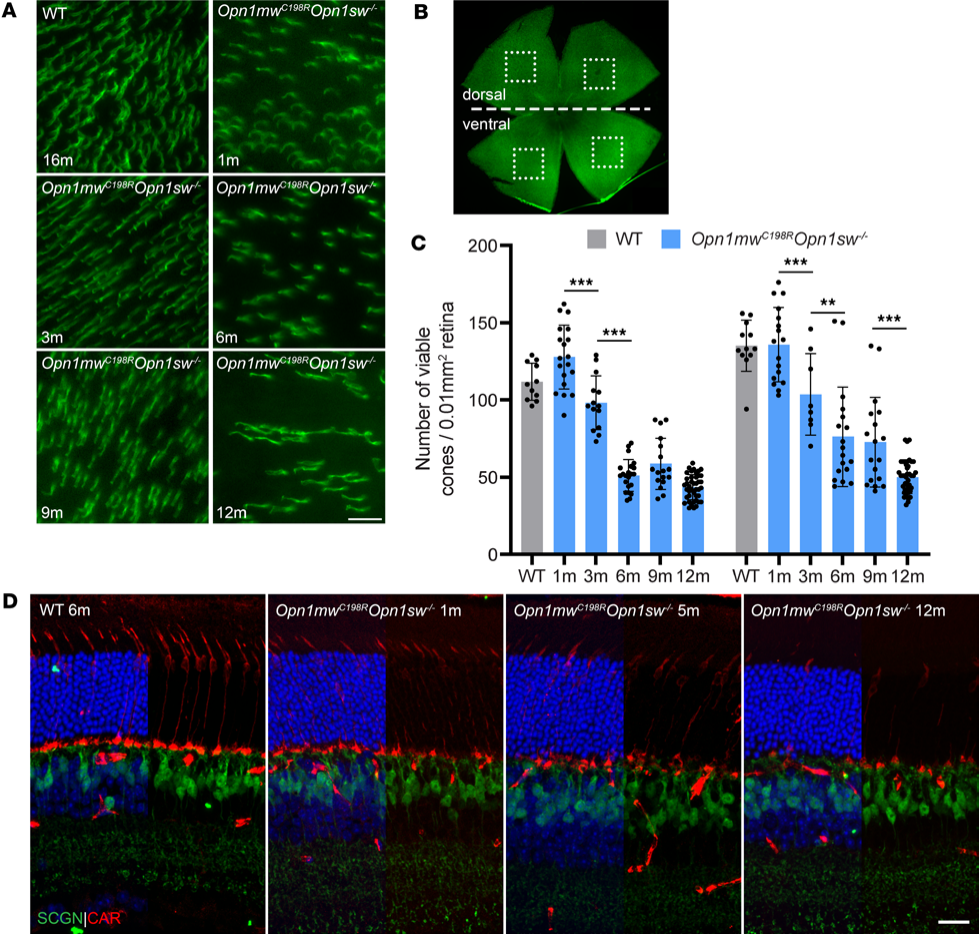
*Opn1mw^C198R^Opn1sw^–/–^* cones exhibit shortened or absent outer segments and undergo degeneration. (**A**) Representative images of *Opn1mw^C198R^* *Opn1sw^–/–^* retinal flat mounts at 1, 3, 6, 9, and 12 months, as well as a WT retinal flat mount at 16 months stained with PNA to show cone viability. Scale bar = 10 μm. (**B**) Cone density was determined by analyzing the number of PNA^+^ cells in 4 different regions (2 dorsal, 2 ventral) from retinal flat mounts, as indicated by the squares that overlie the image. (**C**) Quantification of cone density from the dorsal (left) and ventral (right) regions of WT flat mounts at 12 months (*n* = 12 dorsal/12 ventral) and *Opn1mw^C198R^* *Opn1sw^–/–^* retinal flat mounts at 1 (*n* = 19/17), 3 (*n* = 14/8), 6 (*n* = 22/18), 9 (*n* = 17/18), and 12 months (*n* = 42/42). Each bar represents the number of PNA^+^ cells counted per 0.01 mm^2^ of retinal flat mount surface area. Data shown are the average ± SD, 2-way ANOVA (**P < 0.002, ***P < 0.001). (**D**) IHC of 6-month WT and 1-, 5-, and 12-month *Opn1mw^C198R^* *Opn1sw^–/–^* retinal cross sections stained with antibodies against cone arrestin (CAR, red) and secretagogin (SCGN, green). Scale bar = 20 μm.

Cone viability was assessed by peanut agglutinin (PNA) staining of *Opn1mw^C198R^* *Opn1sw^–/–^* retinal whole mounts at 1, 3, 6, 9, and 12 months of age (Figure 2, A–C) as described previously (27). Previous studies have shown that cone density in the mouse retina is between 10,000 and 14,000 cones/mm^2^ of retina, with no statistical difference between young and aged WT mice (30–32). At 1 month of age, we observed a comparable cone density in the dorsal and ventral regions of *Opn1mw^C198R^* *Opn1sw^–/–^* retinas compared to WT (*n* = 8 mice dorsal, *n* = 7 ventral). However, by 3 months of age, there was a 23% decrease in cone viability (*n* = 8 mice dorsal, *n* = 6 ventral; *P* < 0.05). We also observed another large decrease in cone viability between 3 and 6 months of age, with approximately 50% of dorsal cones (*n* = 8 mice) and 25% of ventral cones (*n* = 7 mice; *P* < 0.05) degenerating by 6 months. Cone viability continued to diminish between 6 and 12 months of age by 14.8% in the dorsal (*n* = 6 mice, *P* < 0.05) and 34.2% in the ventral retina (*n* = 6 mice, *P* < 0.05). However, it is important to note that a large number of cones still survive and remain viable in 1-year-old *Opn1mw^C198R^* *Opn1sw^–/–^* mice.

Cone morphology was then assessed by cone arrestin (CAR) labeling of *Opn1mw^C198R^* *Opn1sw^–/–^* cross sections at 1, 5, and 12 months of age (Figure 2D). In WT mice, CAR staining extended from the synaptic terminal and all the way into the COS. In contrast, CAR labeling was observed only in synaptic terminals, axons, cell bodies, and ISs of *Opn1mw^C198R^* *Opn1sw^–/–^* cones, but not in COSs, verifying markedly shortened or absent COSs. Additionally, fewer CAR^+^ cells were present in 5- and 12-month-old cross sections compared with 1-month-old *Opn1mw^C198R^* *Opn1sw^–/–^* eyes, further validating cone degeneration with age. Strikingly, no apparent abnormalities in *Opn1mw^C198R^* *Opn1sw^–/–^* cone bipolar cells were observed up to 10 months of age, as indicated by IHC staining of the cone bipolar cell-specific protein secretagogin (SCGN) (Figure 2D) or by immunoblot analysis (Supplemental Figure 7). These data demonstrate that although cones in *Opn1mw^C198R^* *Opn1sw^–/–^* retinas lack proper COSs, a large number of cones retain ISs and are viable, making them promising targets for gene therapy.

### Structural and functional rescue of Opn1mw^C198R^ Opn1sw^–/–^ mice following gene therapy at 1 or 3 months of age.

**Figure 3:**
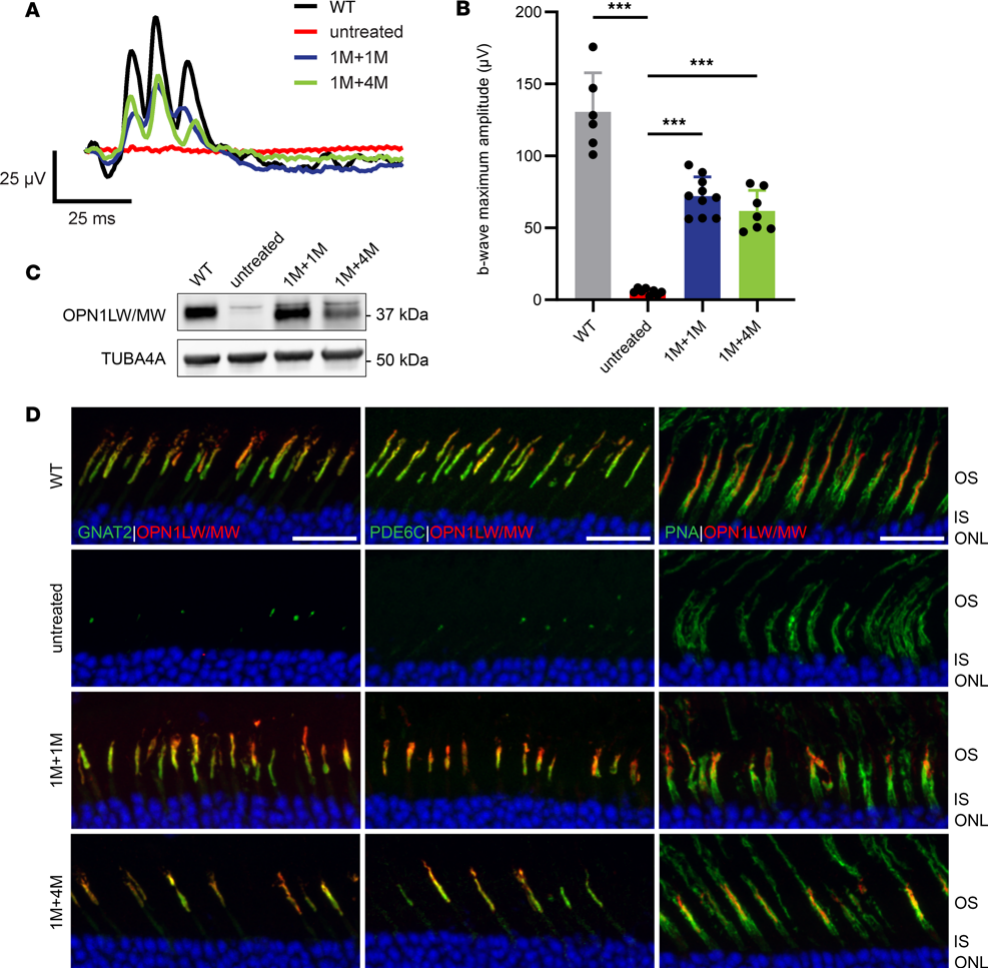
Administration of gene therapy at 1 month of age results in functional and structural rescue of *Opn1mw^C198R^* *Opn1sw^–/–^* cones. (**A**) Representative waveforms and (**B**) b-wave maximum amplitudes of WT (black; *n* = 6 eyes) photopic (white light) ERG, as well as untreated (red; *n* = 8) and 1-month treated *Opn1mw^C198R^* *Opn1sw^–/–^* mice at 1 (1M+1M, blue; *n* = 10) and 4 (1M+4M, green; *n* = 7) months postinjection following L-cone ERG with red (long-wavelength) light at 25 cd●s/m^2^. Data shown are the average ± SD, 1-way ANOVA (***P < 0.001). (**C**) Western blot analysis of WT, untreated, and treated *Opn1mw^C198R^* *Opn1sw^–/–^* retinal lysate at 1 (1M+1M) and 4 (1M+4M) months postinjection showing levels of L-/M-opsin. TUBA4A was used as a loading control. (**D**) Representative IHC images of WT, untreated, and treated *Opn1mw^C198R^* *Opn1sw^–/–^* cross sections labeled with antibodies against L-/M-opsin, GNAT2 (left panels), PDE6C (middle panels), and PNA (right panels). L-/M-opsin, GNAT2, and PDE6C were either undetectable or severely reduced in *Opn1mw^C198R^* *Opn1sw^–/–^* retinas, and treatment rescued their expression and localization to the COS. Scale bar = 20 μm.

**Figure 4:**
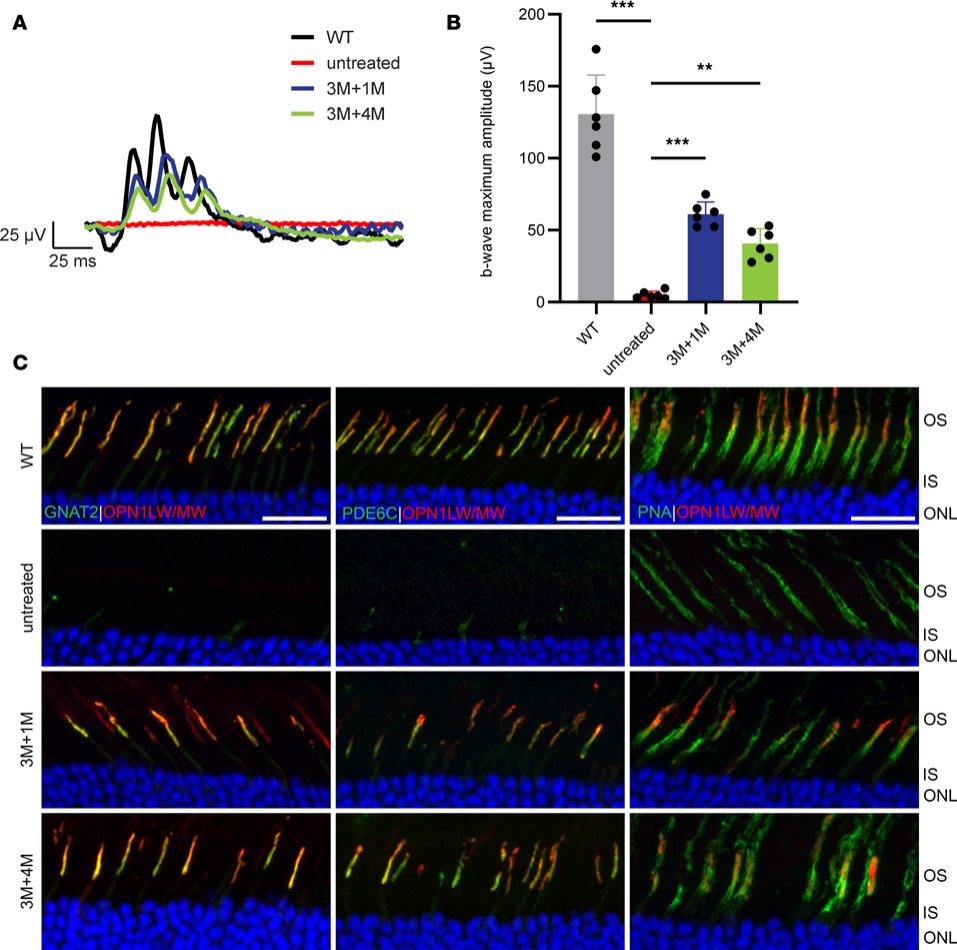
*Opn1mw^C198R^Opn1sw^–/–^* cones display structural and functional rescue when treated at 3 months of age. (**A**) Representative waveforms and (**B**) b-wave maximum amplitudes of WT (black; *n* = 6 eyes) photopic (white light) ERG, as well as untreated (red, *n* = 6) and 3-month treated *Opn1mw^C198R^* *Opn1sw^–/–^* mice at 1 (3M+1M, blue; *n* = 6) and 4 (3M+4M, green; *n* = 6) months postinjection following M-cone ERG with green (medium-wavelength) light at 25 cd●s/m^2^. Data shown are the average ± SD, 1-way ANOVA (**P < 0.002, ***P < 0.001). (**C**) Representative IHC images of WT, untreated, and 3-month treated *Opn1mw^C198R^* *Opn1sw^–/–^* cross sections labeled with antibodies against L-/M-opsin, GNAT2 (left panels), PDE6C (middle panels), and PNA (right panels). Scale bar = 20 μm.

As described above, our data demonstrate that OPN1MW^C198R^ mutant opsin is undetectable in *Opn1mw^C198R^* *Opn1sw^–/–^* retinas and that cone photoceptors in these mice lack proper COS development with abolished cone function. These phenotypes are very similar to those observed in our previously studied DKO mice, a model for BCM with deletion mutations. We next sought to determine whether gene augmentation therapy could rescue cones in *Opn1mw^C198R^* *Opn1sw^–/–^* mice. An AAV5 vector expressing human *OPN1LW* or *OPN1MW* under the control of a cone-specific PR2.1 promoter was administered into the subretinal space of *Opn1mw^C198R^* *Opn1sw^–/–^* eyes at 1 and 3 months of age. The contralateral eyes served as uninjected controls. Cone function was assessed by long-wavelength–mediated (*OPN1LW*) or medium-wavelength–mediated (*OPN1MW*) ERG at 1 and 4 months postinjection to determine the efficacy of gene therapy. We found that when injected at 1 (Figure 3, A and B) and 3 (Figure 4, A and B) months of age, gene augmentation rescued cone function when mice were subjected to ERG at 1 and 4 months postinjection. In 1-month PR2.1-*OPN1LW*–treated animals, the average b-wave maximum amplitude at the highest tested light intensity of 25 cd●s/m^2^ was 72.2 μV ± 13.3 μV (*n* = 10) at 1 month postinjection (1M+1M) and 61.8 μV ± 14.3 μV (*n* = 7) at 4 months postinjection (1M+4M), whereas untreated *Opn1mw^C198R^* *Opn1sw^–/–^* cones displayed no function (Figure 3, A and B). Similarly, at the same light intensity, 3-month treated animals with PR2.1-*OPN1MW* showed an average b-wave maximum amplitude of 61.1 μV ± 8.6 μV (*n* = 6) at 1 month postinjection (3M+1M) and 40.7 μV ± 10.4 μV (*n* = 6) at 4 months postinjection (3M+4M) (Figure 4, A and B). These ERG amplitudes ranged between 30% and 55% of those observed in WT mice.

Cone photoreceptor morphology and cone phototransduction proteins were also analyzed by IHC at these same time points to evaluate the level of structural rescue. Retinal cross sections of untreated and treated *Opn1mw^C198R^* *Opn1sw^–/–^* cones were stained with PNA and antibodies labeling L-/M-opsin, GNAT2, and PDE6C. L-/M-opsin, PDE6C, and GNAT2 expression was undetectable in untreated *Opn1mw^C198R^* *Opn1sw^–/–^* retinal cross sections. In contrast, treated *Opn1mw^C198R^* *Opn1sw^–/–^* cones exhibited elaborated COSs, displayed ample expression of L-/M-opsin to the COS, and showed replenishment of the COS phototransduction proteins GNAT2 and PDE6C when treated with gene therapy at 1 (Figure 3D) and 3 (Figure 4C) months of age and analyzed at 1 and 4 months postinjection. Immunoblot analysis of retinal lysate from 1M+1M treated *Opn1mw^C198R^* *Opn1sw^–/–^* retinas also revealed restoration of L-/M-opsin expression following administration of gene therapy (Figure 3C). Together, these data demonstrate that when treated at 1 or 3 months of age, gene augmentation can provide structural and functional rescue to *Opn1mw^C198R^* *Opn1sw^–/–^* cones.

### Assessing long-term rescue in treated Opn1mw^C198R^ Opn1sw^–/–^ cones.

**Figure 5:**
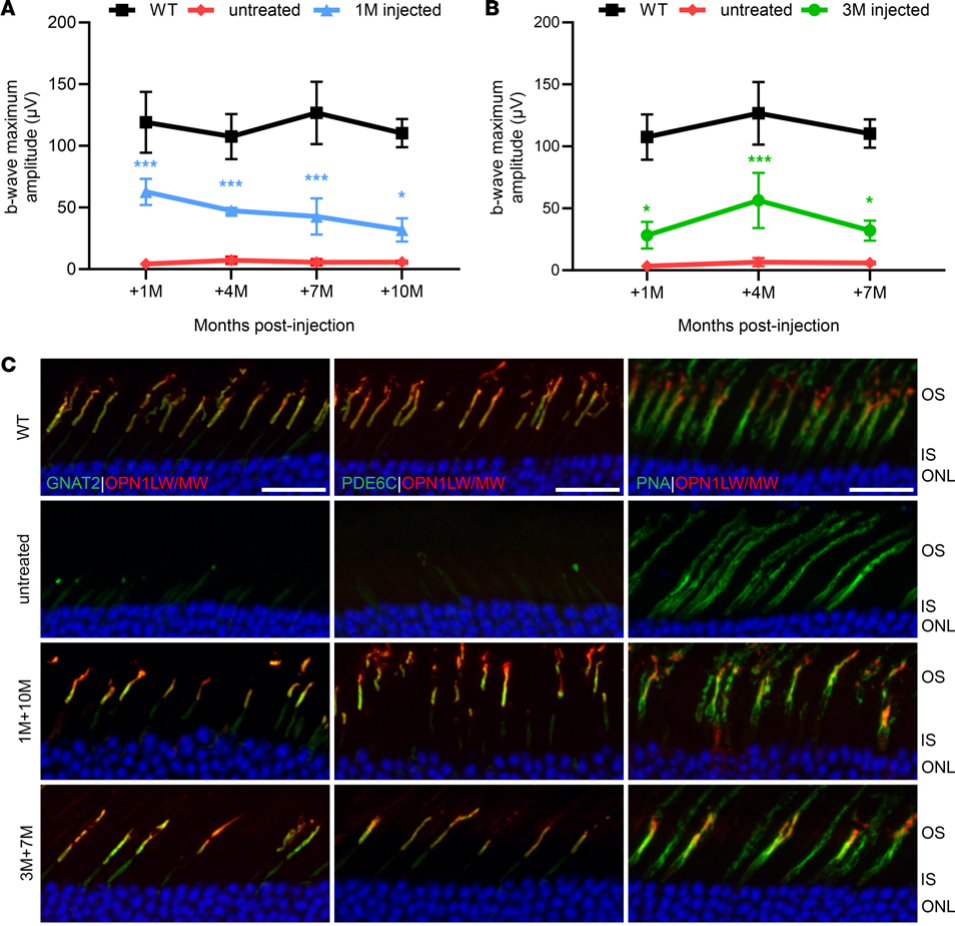
Longevity of functional and structural rescue of *Opn1mw^C198R^* *Opn1sw^–/–^* cones treated at 1 and 3 months of age. (**A** and **B**) Time course study of the b-wave maximum amplitudes following L-cone ERG with red (long-wavelength) light at 25 cd●s/m^2^ of untreated *Opn1mw^C198R^* *Opn1sw^–/–^* mice (*n* = 4) and those treated at (**A**) 1 month and ERG tested at 1, 4, 7, and 10 months postinjection (blue; *n* = 4) and (**B**) *Opn1mw^C198R^* *Opn1sw^–/–^* mice treated at 3 months and ERG tested at 1, 4, and 7 months postinjection (green; *n* = 4). WT (black; *n* = 3) mice were subjected to ERG with photopic (white light) conditions as a control. Data shown are the average ± SD, 2-way ANOVA (*P ≤ 0.05, ***P < 0.001). (**C**) Representative IHC images of WT, untreated, and 3-month treated *Opn1mw^C198R^* *Opn1sw^–/–^* cross sections labeled with antibodies against L-/M-opsin (red) and GNAT2 (left panels, green), PDE6C (middle panels, green), and PNA (right panels, green). Scale bar = 20 μm.

To determine the rescue longevity following administration of gene therapy, we assessed cone function and structure in 1- (Figure 5A) and 3-month (Figure 5B) PR2.1-*OPN1LW* treated *Opn1mw^C198R^* *Opn1sw^–/–^* mice at 1, 4, and 7 months postinjection. Due to the retained rescue in 1M+7M animals, we also assessed their retinal function and morphology at 10 months postinjection (Figure 5A). We observed that while cone function declined from 4 months to 7 months, as well as between 7 and 10 months for 1-month treated mice, rescue was still evident at the date of sacrifice. Importantly, at these endpoints, we still saw a large number of L-/M-opsin^+^ cones as indicated by PNA staining (Figure 5C, right panels) that expressed ample levels of both GNAT2 (Figure 5C, left panels) and PDE6C (Figure 5C, middle panels).

### Diminished rescue efficacy in Opn1mw^C198R^ Opn1sw^–/–^ mice treated at 5 months of age.

**Figure 6:**
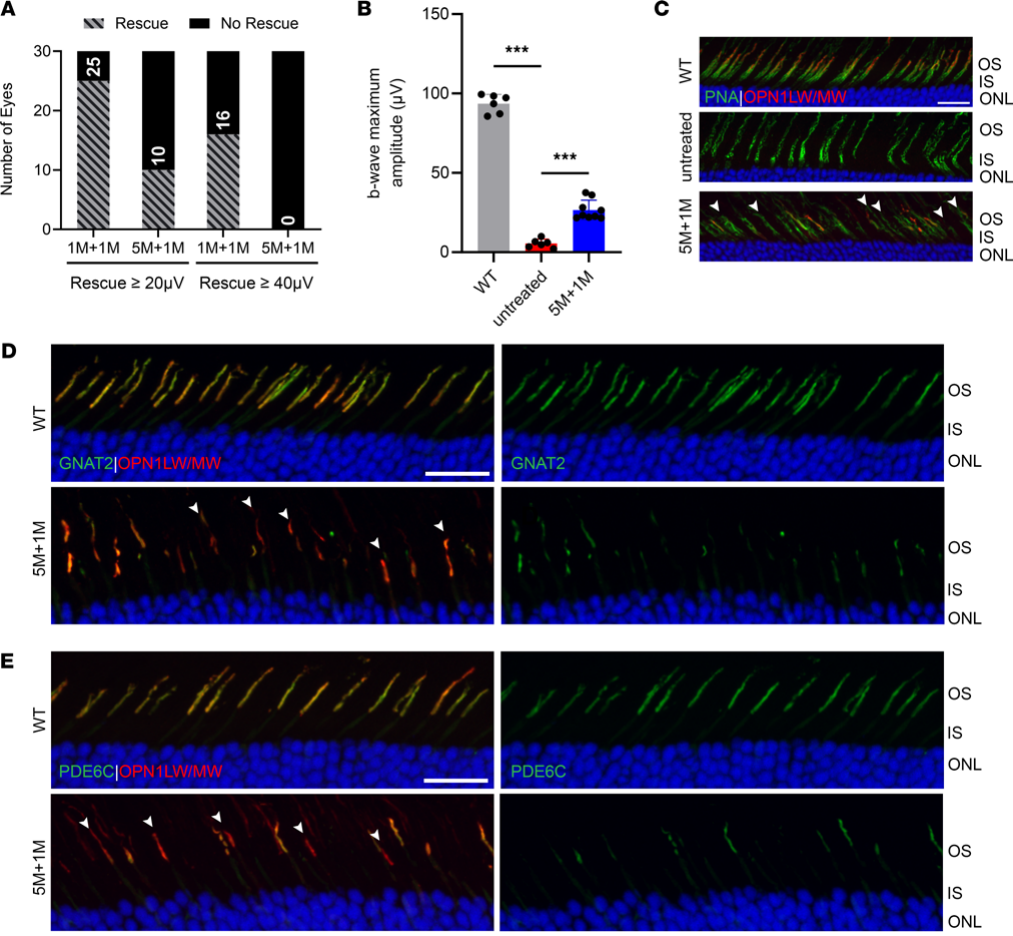
Gene augmentation therapy shows reduced rescue efficacy of cone structure and function in 5-month-old treated *Opn1mw^C198R^* *Opn1sw^–/–^* animals. (**A**) Rescue efficiency of *Opn1mw^C198R^* *Opn1sw^–/–^* mice treated at 1 (*n* = 30 eyes) and 5 (*n* = 30 eyes) months of age, determined by functional cone rescue as a b-wave maximum amplitude above 20 μV (left) or 40 μV (right). (**B**) b-wave maximum amplitudes of WT (black, *n* = 6) photopic (white light) ERG, as well as untreated (red, *n* = 6) and 5M+1M treated *Opn1mw^C198R^* *Opn1sw^–/–^* mice (blue, *n* = 9) following L-cone ERG with red (long-wavelength) light at 25 cd●s/m^2^. Data shown are the average ± SD, 1-way ANOVA (***P < 0.001). (**C**) Representative IHC images of 6-month untreated and 5M+1M treated *Opn1mw^C198R^* *Opn1sw^–/–^* cross sections labeled with PNA (green) and an antibody against L-/M-opsin (red). Viable cones positive for PNA staining but lacking L-/M-opsin expression are indicated by arrows. Scale bar = 20 μm. (**D** and **E**) Representative IHC images of WT and treated 5M+1M *Opn1mw^C198R^* *Opn1sw^–/–^* cross sections labeled with antibodies against L-/M-opsin (red) and (**D**) GNAT2 (green) or (**E**) PDE6C (green). Cones positive for L-/M-opsin while showing severely reduced GNAT2 and PDE6C are indicated by arrows. Scale bar = 20 μm.

At 6 months of age, 54% of *Opn1mw^C198R^* *Opn1sw^–/–^* cones remained that contained intact ISs, suggesting the possibility of gene therapy at 5 months. However, we found that delivery of gene therapy to 5-month old *Opn1mw^C198R^* *Opn1sw^–/–^* eyes resulted in a poor outcome. We compared 2 cohorts of mice, 1 treated at 1 month of age (*n* = 30) and another treated at 5 months (*n* = 30). When we compared cone ERG responses at 1 month postinjection, we observed that 83% of 1M+1M mice showed a b-wave maximum amplitude above 20 μV, whereas only 33% of 5M+1M mice showed the same rescue (Figure 6A, left panel). Furthermore, not a single 5M+1M treated eye from this cohort showed rescue above 40 μV, though approximately 53% of 1M+1M eyes showed rescue at or above this threshold (Figure 6A, right panel). The average b-wave maximum amplitude at 25 cd●s/m^2^ from rescued eyes (*n* = 9 eyes) was only 26.6 μV ± 6.2 μV for 5M+1M mice (Figure 6B), which was significantly lower than that observed for 1M+1M eyes.

When we analyzed images of cone structure and protein localization in 5-month treated eyes, we found that only approximately one-third of the remaining cones at this age (indicated by PNA staining) expressed L-/M-opsin (Figure 6C). Moreover, in L-/M-opsin^+^ cones, the expression levels of GNAT2 (Figure 6D) and PDE6C (Figure 6E) were severely reduced compared with what was observed in *Opn1mw^C198R^* *Opn1sw^–/–^* animals treated at 1 or 3 months of age. In combination with our results from treatment at younger ages, these data suggest that gene augmentation therapy is efficacious in *Opn1mw^C198R^* *Opn1sw^–/–^* animals, providing evidence that treatment must be initiated early in order to achieve the desired structural and functional rescue.

## Discussion

In the current study, we establish and characterize a mouse model of BCM for patients carrying the most common missense mutation in cone opsin, C203R. Taken together, our data demonstrate that cone function and structure in *Opn1mw^C198R^* *Opn1sw^–/–^* mice resemble those of L- and M-cones in BCM patients with a C203R mutation, representing a suitable model for studying the disease mechanism of BCM and for developing gene therapy strategies. We demonstrate that *Opn1mw^C198R^* *Opn1sw^–/–^* mice exhibit normal rod function at P30 but lack photopic ERG responses. Moreover, *Opn1mw^C198R^* *Opn1sw^–/–^* cones display noticeably shortened or absent outer segments, mimicking the retinal phenotype seen in BCM patients with the same mutation. Remarkably, gene augmentation therapy led to robust rescue of cone function and outer segments when mice were treated at 1 or 3 months of age, and the therapeutic effect lasted for at least 7 months posttreatment. A caveat, however, is that the efficacy of gene therapy is severely reduced in *Opn1mw^C198R^* *Opn1sw^–/–^* mice treated at 5 months of age, despite more than 50% of viable cones remaining at the time of treatment. To our knowledge, this is the first characterization of a mouse retinal phenotype caused by a cone opsin missense mutation and is the first demonstration that gene augmentation therapy can rescue cone photoreceptor function and structure in such a model. Our research also suggests that there may be fundamental differences between rods and cones in regard to management of cellular stress elicited by expression of misfolded or mislocalized proteins.

Previous research has shown that a C203R mutation in L-/M-opsin results in protein misfolding, ER retention, loss of ability to absorb light, and a reduced half-life when overexpressed in cell culture (15). Based on these findings, it was initially postulated that expression of mutant C203R protein would be toxic to cone photoreceptors due to disruption of ER homeostasis, similar to what has been observed for various rhodopsin missense mutations linked to adRP (21, 33, 34). We were unable to note any obvious toxicity to cones caused by expression of OPN1MW^C198R^ mutant opsin as *Opn1mw^C198R^* *Opn1sw^–/–^* cones degenerated at a similar rate as cones in our previously characterized cone opsin deletion model of BCM (27).

Use of adeno-associated virus–mediated (AAV-mediated) gene augmentation to deliver functional rhodopsin in the clinically relevant *Rho^P23H^* knockin mice, which carry the most common mutation associated with adRP, did not demonstrate any beneficial structural or functional rescue in rods (35). Unfortunately, rods carrying the burden of RHO^P23H^ seem to be unable to rid themselves completely of mutant P23H protein, with a significant amount of misfolded P23H rhodopsin eluding degradation and trafficking to the rod outer segment, resulting in myriad consequences for rod photoreceptor function and health (36–45). Therefore, the level of functional and structural rescue in 1- and 3-month treated *Opn1mw^C198R^* *Opn1sw^–/–^* cones was both surprising and intriguing. Upon closer examination of *Opn1mw^C198R^* *Opn1sw^–/–^* cones, we were unable to observe any expression of OPN1MW^C198R^ mutant protein by IHC or immunoblot at any age. Though reduced compared with WT, levels of *Opn1mw^C198R^* mRNA were detectable, suggesting that regulation of mutant OPN1MW^C198R^ expression might occur at both the mRNA and protein level. While much remains unknown, when we compare our data to studies with P23H rhodopsin, it is clear that there are obvious differences between the fate of RHO^P23H^ in rods and OPN1MW^C198R^ in cones. Regardless of these differences, we can still look to models of P23H rhodopsin for future investigation. It is possible that cones regulate expression of misfolded cone opsin through similar pathways as rods regulate P23H rhodopsin, with previous studies demonstrating that *Rho^P23H^* mRNA is degraded through mRNA decay and ribosome quality control, while translated P23H rhodopsin is degraded through ER-associated degradation (36, 46). Another possibility is that mutant mRNA is subjected to miRNA-mediated decay, as previous studies have shown that miRNAs play a crucial role in photoreceptor homeostasis, function, and survival (47). Nevertheless, mouse cones appear to boast the incredible ability to mitigate the burden of expressing C203R misfolded cone opsin protein by successfully degrading it, preventing stress and disruption of ER homeostasis. Together, our results raise an interesting question as to whether gene augmentation therapy will also benefit cones expressing other identified, and presumably misfolded, cone opsin missense mutations, such as N94K and W177R (48).

In *Opn1mw^C198R^* *Opn1sw^–/–^* mice treated at 5 months of age, only approximately one-third of remaining cones were found to express AAV-mediated *OPN1LW* when analyzed at 1 month postinjection. Replenishment of GNAT2 and PDE6C was also diminished in OPN1LW^+^ cones compared with the abundant expression we observed in 1- and 3-month treated cones, suggesting inefficient transcription or translation of other key phototransduction proteins, or dysfunctional ciliary targeting of COS proteins to their proper compartment. Based on these data, we believe there are a few possibilities that could result in the observed reduction in therapy efficacy: A) the remaining aged *Opn1mw^C198R^* *Opn1sw^–/–^* cones have lost the ability to be transduced by AAV, B) the cones are transduced by AAV but are unable to produce the required expression level of the transgene in combination with synthesizing adequate levels of other COS proteins, or C) the connecting cilium is damaged in older cones, resulting in dysfunctional ciliary targeting of COS proteins. These hypotheses are not mutually exclusive. Therefore, elucidating the defects in aged BCM cones that render them untreatable is critical in order to expand the therapeutic window. Future experiments will include detailed characterization of ultrastructural changes, as well as profiling differences in gene expression between young and aged *Opn1mw^C198R^* *Opn1sw^–/–^* cones.

The robust rescue observed in *Opn1mw^C198R^* *Opn1sw^–/–^* animals treated at 1 and 3 months of age also prompts us to compare the rate of cone degeneration and the therapeutic window for the 2 most common causes of BCM, including the C203R model of BCM in the current study, as well as our earlier published DKO mouse model for patients with BCM with deletion mutations (27). Importantly, studies evaluating the retinal and foveal structures of patients with BCM carrying either of these mutations have demonstrated that patients with a C203R missense mutation, despite expressing a “toxic” mutant protein, actually exhibit decades-longer persistence of outer nuclear layer thickness and show a slower rate of development of inner segment/outer segment defects compared with patients carrying large deletion mutations (21). These findings suggest that there may be a much wider window of opportunity to treat patients with BCM with a C203R missense mutation. In our study, we find that *Opn1mw^C198R^* *Opn1sw^–/–^* cones do not degenerate at a noticeably accelerated rate because of expression of misfolded OPN1MW^C198R^ protein, and possess at least an equivalent, if not wider, therapeutic window compared with DKO mice. Furthermore, we see that gene therapy is efficacious when administered early in both models, but less effective in 5M+1M treated animals, emphasizing the importance of dissecting molecular changes in aged cones. Nevertheless, a side-by-side comparison study treating these 2 BCM animal models with the same batch of AAV at older ages is needed to accurately determine differences in the therapeutic window between the two. Thus, our results from the current study, as well those from our DKO animal model of BCM, are consistent with findings reported for corresponding human patients with BCM.

In summary, we observe efficient structural and functional rescue in *Opn1mw^C198R^* *Opn1sw^–/–^* cones following administration of AAV-mediated gene augmentation therapy. Additionally, these cones appear to possess the amazing ability to protect themselves from cellular stressors caused by expression of misfolded OPN1MW^C198R^ mutant opsin protein in addition to managing the burden of degrading other COS proteins that are left without an outer segment to reside within. Further work is needed to decipher molecular changes occurring between young and aged cones affected by BCM in order to continue developing new gene therapy strategies, as well as to extend the therapeutic window in treating BCM.

## Methods

### Animals.

*Opn1mw^C198R^* mice were sponsored by the BCM Families Foundation to be generated at Charles River in Europe and gifted to our lab. More information on these mice can be found at Jackson Laboratory (Strain: 031385). We crossed *Opn1mw^C198R^* mice to C57BL/6J mice (Jackson Laboratory, Strain: 000664) for 5 generations to eliminate any potential mosaics and off-target effects from CRISPR/Cas, then bred them to homozygous (females) and hemizygous (males) for characterization. *Opn1mw^C198R^* *Opn1sw^–/–^* mice were then generated by crossing *Opn1mw^C198R^* and *Opn1sw^–/–^* mice (Jackson Laboratory, Strain: 032295). Genotyping of *Opn1mw^C198R^* mice was performed by the protocol below or by service provided at Transnetyx. Genotyping for *Opn1sw^–/–^* mice was performed as previously described (49). Both male and female mice were used in all experiments. For *Opn1mw^C198R^* knockin mice, we used both homozygous *Opn1mw^C198R/C198R^* females and hemizygous *Opn1mw*^C198R/Y^ males. All mice used in this study were maintained in the West Virginia University and University of Florida Health Science Center Animal Care Service Facilities under 12-hour light/12-hour dark cycles with food and water provided ad libitum.

### Genotyping of Opn1mw^C198R^ mice.

DNA was extracted from mouse tails using DNeasy Blood and Tissue Kit (QIAGEN) according to the manufacturer’s protocol. The PCR primers are forward GACAAGGATTTACCAAAATCCTACT and reverse CAGAGCACGATGATGCTGAGTGGGA. PCR was performed with Phusion HS (Thermo Fisher Scientific) using the following conditions: 98°C for 30 seconds, then 35 cycles of 98°C for 10 seconds, 60°C for 10 seconds, 72°C for 40 seconds, followed by a final extension of 72°C for 5 minutes. PCR product was then cleaned using a DNA Clean & Concentrator-5 kit (Zymo Research), followed by digestion with ApaI restriction enzyme, and analyzed by 2.5% agarose gel electrophoresis. The PCR produces a 365 bp band. An ApaI restriction site is engineered right next to C198R mutation, and ApaI digestion will produce 2 bands at 266 bp and 99 bp bands for mutant mice to differentiate from WT mice. We also keep in mind that the mutated allele is on chromosome X.

### Cell culture.

HEK293T cells (American Type Culture Collection) were maintained in DMEM/F-12 media (Gibco, Thermo Fisher Scientific) supplemented with 10% FBS (Gibco, Thermo Fisher Scientific) and 1% penicillin-streptomycin (Gibco, Thermo Fisher Scientific) in an incubator set to 37°C with 5% CO_2_. Cells were plated in a 6-well plate and allowed to grow for 24 hours prior to transfection. Cells were transfected using constructs with a chicken β-actin promoter encoding human OPN1MW-HA or OPN1MW^C203R^ with TransIT-LT1 (Mirus Bio) as directed by the manufacturer’s protocol. Cells were then collected 48 hours following transfection using ice-cold 1× PBS before Western blot analysis.

### RNA extraction, cDNA synthesis, and quantitative real-time PCR.

Total RNA was extracted from WT, *Opn1mw^C198R^*, and *Opn1mw^C198R^* *Opn1sw^–/–^* mouse retinas using a Micro-to-Midi total RNA purification system (Invitrogen). DNase treatment before the reverse transcription was performed to prevent amplification from genomic DNA contamination. First-strand cDNA was generated with SuperScript II reverse transcriptase (Invitrogen). Real-time PCR was performed with iQ SYBR green supermix (Bio-Rad) according to manufacturer’s instructions. The sequences of the primers were TGTGTCATTGAAGGCTACAT (forward) and TGACCATGAGGACCATCATA (reverse), derived from different exons of *Opn1mw* surrounding the C198R mutation. The PCR products were analyzed by agarose gel electrophoresis, followed by Sanger sequencing to confirm the presence of a C198R mutation.

### AAV vectors.

The AAV vectors used in this study contain a PR2.1 promoter (50) that drives expression of human *OPN1LW* or *OPN1MW* (PR2.1-hOPN1LW, PR2.1-hOPN1MW). All vectors were packaged in AAV5 and purified according to previously published methods at the University of Florida Ocular Gene Therapy Core (51).

### Subretinal injection.

Prior to injection, mouse eyes were dilated using Tropi-Phen drops (Pine Pharmaceuticals). Mice were then anesthetized by intramuscular injection using ketamine (80 mg/kg) and xylazine (10 mg/kg) in sterile PBS. The viral vector for injection was prepared by adding fluorescein dye (0.1% final concentration) to AAV at a concentration of 1 × 10^10^ vector genomes per microliter. A 25-gauge needle was then used to introduce a small hole at the edge of the cornea. Next, a transcorneal subretinal injection was achieved by inserting a 33-gauge blunt-end needle attached to a 5 μL Hamilton syringe containing 1 μL of prepared AAV and injecting into the subretinal space. The injection bleb was visualized by the spread of fluorescence in the subretinal space. Immediately after injection, eyes were treated with Neomycin/Polymixin B Sulfates/Bacitracin Zinc ophthalmic ointment (Bausch & Lomb), and antisedan (Orion Corporation) was injected intraperitoneally to reverse anesthesia.

### ERG.

Prior to ERG testing, all eyes were dilated using Tropi-Phen drops. Animals were then anesthetized with isoflurane (5% in 2.5% oxygen) for 3–5 minutes and subsequently placed on a heated stage (37°C) with a nose cone supplying isoflurane (1.5% in 2.5% oxygen) throughout testing. Eyes were lubricated with GenTeal gel (0.3% hypromellose), and silver wire electrodes were placed above the cornea. A ground electrode was placed in the animals’ tails, and a reference electrode was placed between the ears subcutaneously. ERG recordings were conducted using the UTAS Visual Diagnostic System with BigShot Ganzfeld, UBA-4200 amplifier and interface, and EMWIN software (version 9.0.0, LKC Technologies). For scotopic ERGs, mice were dark-adapted overnight. Scotopic (rod) ERGs were performed in the dark with flashes of white light at 5 different light intensities (–3.6, –3, –0.8, 0, and 0.4 log cd●s/m^2^). Photopic (cone) responses were performed with a 30 cd/m^2^ white background light after light adapting mice for 5 minutes to saturate rods. Cone-mediated ERG responses were collected at increasing light intensities (0.4, 0.7, 0.9, 1.4 log cd●s/m^2^) following stimulation with white light, short- (360 nm), middle- (530 nm), or long- (630 nm) wavelength light.

### Retinal whole mount preparation and assessing cone viability.

After humane euthanasia of our mice, eyes were marked at the dorsal position of the cornea using the Change-a-Tip Deluxe Cautery tool (Braintree Scientific) and enucleated. Next, a 16-gauge needle was used to poke a hole along the edge of the cornea, and the eye was placed in 4% paraformaldehyde (PFA) in 1× PBS. Following a 2-hour incubation at room temperature, the cornea and lens were removed, a radial cut was made at the dorsal position, and the neural retina was dissected away from the eyecup. Retinas were then blocked in 3% bovine serum albumin (BSA) with 0.3% Triton X-100 in 1× PBS for 2 hours at room temperature and then labeled with biotinylated PNA at a 1:500 dilution (Vector Laboratories) in 1% BSA in PBS overnight at 4°C. Following 3 washes in 0.05% Tween 20 in PBS, the retinal whole mounts were incubated overnight with Fluorescein Avidin D at a 1:500 dilution (Vector Laboratories) in PBS. Finally, retinas were washed and 3 additional radial cuts were made at the ventral, temporal, and nasal edges of the retina. The retinal whole mount was flattened beneath a coverslip using ProLong Gold Antifade Mountant (Thermo Fisher Scientific). Whole mounts were imaged using a Leica Fluorescence Microscope LAS X Widefield System. Four images were captured (2 ventral, 2 dorsal) for each retinal whole mount, and PNA^+^ cells from 12 images (*n* = 6, 3 males, 3 females) were counted in an area equivalent to 0.01 mm^2^ of retina for each image using the counting tool in Adobe Photoshop and averaged for the dorsal and ventral regions. When quantifying cone viability with retinal flat mounts, cones within the dorsal or ventral region were compared between different age groups, and only statistical significance from one age to the next was annotated on the graph.

### IHC and frozen retinal cross section preparation.

Mice were humanely euthanized and prior to enucleation, the dorsal position was marked on the cornea using the Change-a-Tip Deluxe Cautery tool. A hole was then poked along the edge of the cornea using a 16-gauge needle, and the eye was incubated in 4% PFA in 1× PBS for 2 hours at room temperature. Then the cornea was cut away from the eye. Next, eyes were washed twice in 1× PBS for 10 minutes and placed in 20% sucrose in 1× PBS overnight at 4°C. The following day, the eyes were incubated in a 50/50 mixture of 20% sucrose in 1× PBS and Tissue-Tek O.C.T. compound (Sakura Finetek USA) for 1 hour at 4°C. Finally, eyes were placed in a cryomold filled with O.C.T. and flash-frozen in a dry ice and ethanol bath. Prior to staining of retinal tissue, 16 μm cross sections were cut using a Leica CM1850 Cryostat and placed on Superfrost plus slides (Thermo Fisher Scientific). A hydrophobic barrier was drawn around the tissue on the slides and then washed with 1× PBS to eliminate O.C.T. compound and hydrate the retinal cross sections. Tissue was then incubated in block buffer containing 3% BSA and 0.3% Triton X-100 for 1 hour at room temperature before addition of primary antibody (see Table 1) diluted in 1% BSA in 1× PBS, which incubated overnight at 4°C. The following day, cross sections were washed with 0.1% Triton X-100 in 1× PBS and secondary antibody (see Table 1; 1:500), and DAPI (1:1,000; Thermo Fisher Scientific) diluted in 1× PBS was added and allowed to incubate for 2 hours at room temperature. After additional washes in 0.1% Triton X-100 in 1× PBS, coverslips were mounted using ProLong Gold Antifade Mountant. Retinal cross sections were imaged using a Nikon C2 confocal microscope and processed using FIJI software (52). Representative images of IHC studies were chosen following at least 3 technical and 3 biological replicates.

### Immunoblot analysis.

Retinas were carefully dissected following humane euthanasia and flash-frozen on dry ice. For analysis by Western blot, each retina was placed in 80 μL of protein extraction buffer containing 0.23 M sucrose, 5 mM Tris-HCl (pH 7.5), and 1× cOmplete protease inhibitor cocktail (MilliporeSigma) and homogenized by sonication (Microson Ultrasonic cell disruptor, 3 pulses 5 seconds at power setting 10). Protein concentration of the retinal lysate was determined using a NanoDrop spectrophotometer (ND-1000, Thermo Fisher Scientific), and 4× Laemmli Sample Buffer (Bio-Rad) with 10% 2-mercaptoethanol was added. Next, 75 μg of total protein was loaded for each sample into a 10% Mini-PROTEAN TGX precast gel (Bio-Rad) and transferred to an Immun-Blot Low Fluorescence PVDF Membrane (Bio-Rad) at 100 V for 1 hour on ice. Membranes were subsequently blocked in Intercept (PBS) Blocking Buffer (LI-COR Biosciences) for 1 hour at room temperature and incubated in primary antibody diluted in a 50/50 mixture of block and wash buffer overnight at 4°C. The following day, membranes were washed in 0.1% Tween 20 in 1× PBS and incubated in secondary antibody (see Table 1) for 1 hour at room temperature. Secondary antibody was diluted in buffer containing 0.05% Tween 20, 10% block buffer, and 0.02% SDS in 1× PBS. After additional washes, blots were imaged using an Odyssey Infrared Imaging System, and protein density was measured according to manufacturer’s instructions (LI-COR Biosciences). Representative immunoblot images were chosen following at least 3 technical and 3 biological replicates.

### Statistics.

All data are presented as the mean ± SD unless otherwise noted, and figure legends contain details about sample size. All analyses were carried out using GraphPad Prism 9 software by unpaired, 2-tailed Welch’s *t* test (2 groups), ordinary 1-way ANOVA with Tukey’s ad hoc test (more than 2 groups), or 2-way ANOVA with Tukey’s ad hoc test for multiple comparisons unless noted differently. *P* ≤ 0.05 was deemed statistically significant.

### Study approval.

All experimental procedures involving animals in this study were approved by and conducted in strict accordance with relevant guidelines and regulations by the Institutional Animal Care and Use Committee at University of Florida and West Virginia University, the Association for Research in Vision and Ophthalmology Statement for the Use of Animals in Ophthalmic and Vision Research, and NIH regulations.

### Data availability.

The data generated and/or analyzed in the current study are included in this article or its Supporting Data Values file. All underlying data relating to this study are available from the corresponding author upon request.

## Author contributions

ERS and WTD designed research; ERS, XM, MEC, RJB, YW, and WTD performed research; ERS, XM, MEC, and WTD analyzed data; and ERS and WTD wrote and edited the manuscript.

## Supplementary Material

Supplemental data

Supporting data values

## Figures and Tables

**Table 1:**
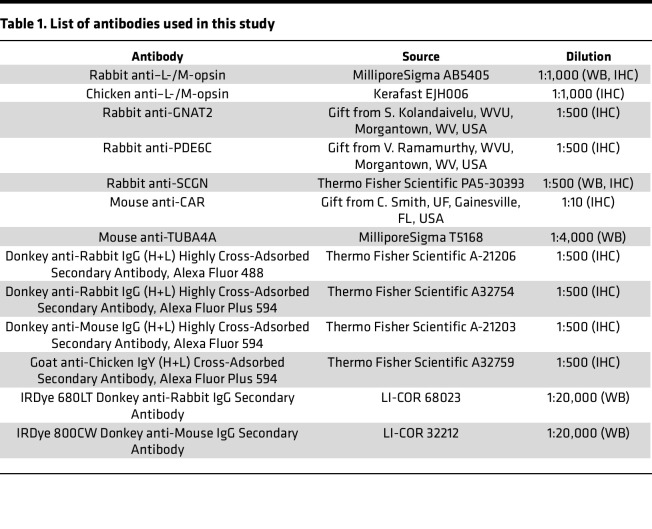
List of antibodies used in this study

## Version 1 12/07/2023

In-Press Preview

## Version 2 01/23/2024

Electronic publication
