# Supplementary material for: Structural and functional rescue of cones carrying the most common cone opsin C203R missense mutation
Source: JCI Insight. 2024 Jan 23;9(2):e172834. doi: 10.1172/jci.insight.172834 (PMC10906232; doi:10.1172/jci.insight.172834)
Supplement: Supplemental data [file jciinsight-9-172834-s073.pdf]

SUPPLEMENTAL MATERIALS

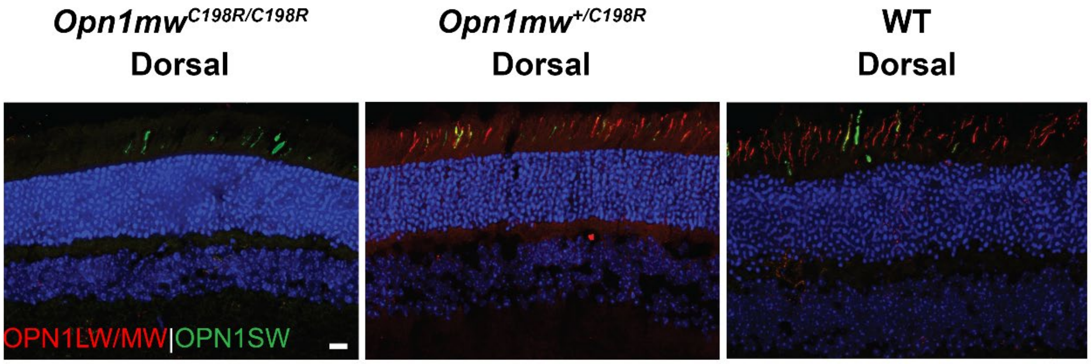

Supplemental Figure 1. M- and S-opsin localization in homozygous and heterozygous *Opn1mw^{C198R}* mice. Representative IHC images of the dorsal region of WT, heterozygous *Opn1mw$^{+/C198R}$* female, and homozygous *Opn1mw^{C198}* cross-sections collected at P30 and stained with antibodies against S-opsin (green) and M-opsin (red). Scale bar = 20μm.

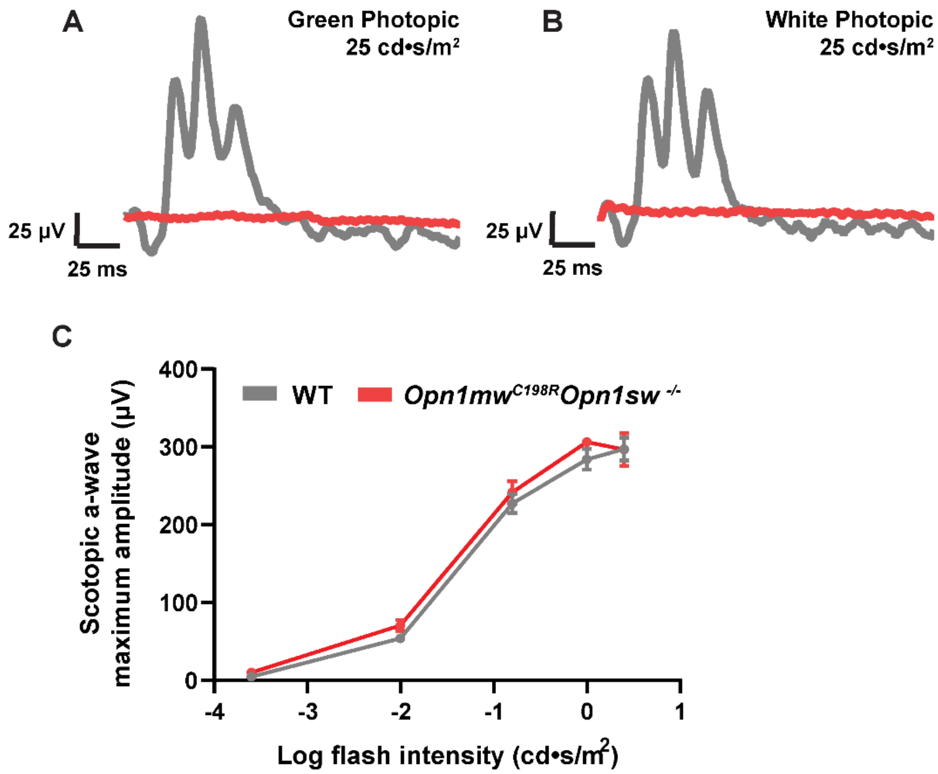

Supplemental Figure 2. ***Opn1mw*^{C198R}*Opn1sw*$^{-/-}$ cones are non-functional while rods are normal. (A-B)** Representative waveforms of P30 WT (grey) and *Opn1mw*^{C198R}*Opn1sw*$^{-/-}$ mice (red) following ERG recordings at 25 cd●s/m^2 under **(A)** middle-wavelength (green) and **(B)** photopic (white) light. **(C)** Sensitivity curve demonstrating the scotopic a-wave maximum amplitude of WT (grey) and *Opn1mw*^{C198R}*Opn1sw*$^{-/-}$ mice (red) at P30 following ERG recordings under dim light conditions at various light intensities (0.0003, 0.01, 0.2, 1.0, and 2.5 cd●s/m^2). Data is represented as mean ± SEM (n=6).

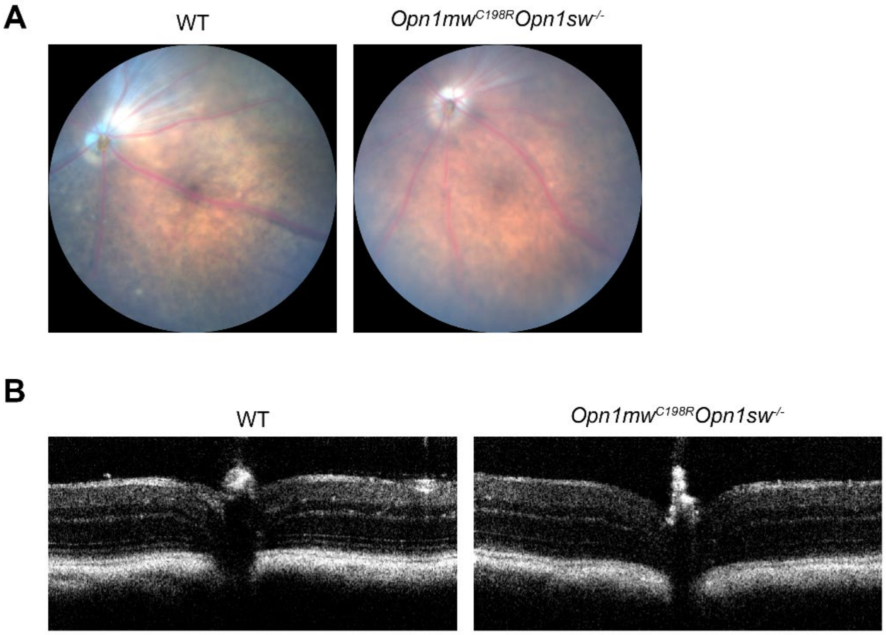

Supplemental Figure 3. Normal morphology of *Opn1mw*^{C198R}*Opn1sw*$^{-/-}$ retinas. (A) Representative bright field fundus images and **(B)** OCT images from 10-month old WT and *Opn1mw*^{C198R}*Opn1sw*$^{-/-}$ mice revealed no gross morphological abnormalities (n=3).

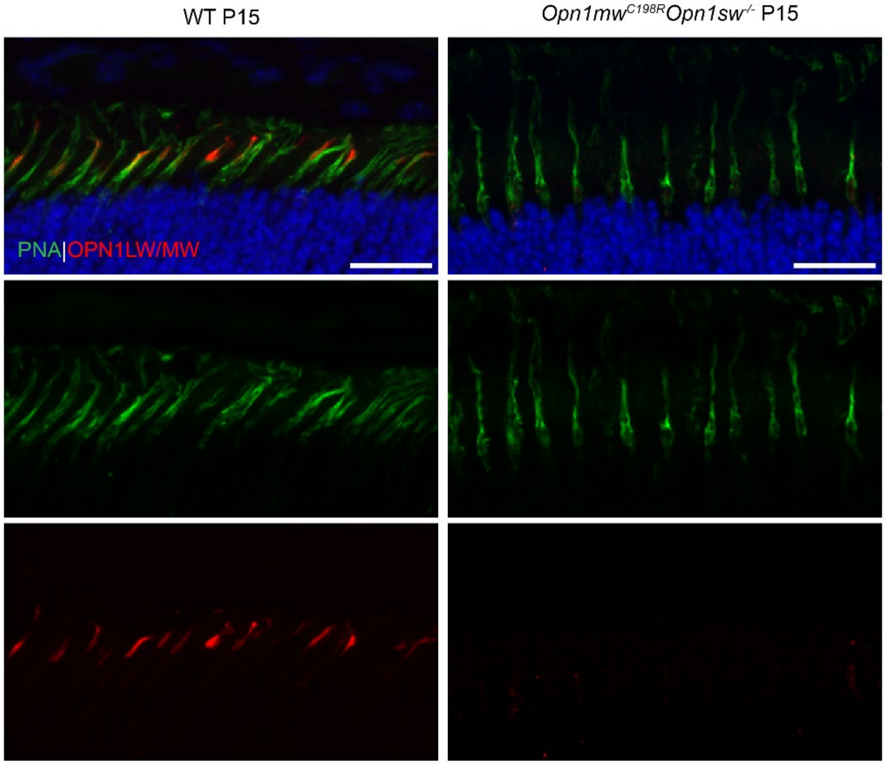

Supplemental Figure 4. *Opn1mw^{C198R}Opn1sw$^{-/-}$* cones lack L/M-opsin expression at P15. Representative IHC images of WT (left) and *Opn1mw^{C198R}Opn1sw$^{-/-}$* (right) cross-sections stained with an antibody against L/M-opsin and with PNA. Scale bar = 20µm.

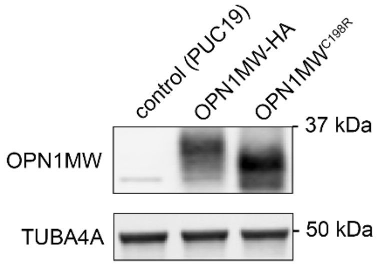

Supplemental Figure 5. Antibody against L/M-opsin recognizes mutant OPN1MW^{C198R}. Western blot analysis of HEK293T cell lysates following single-plasmid transfection with PUC19 (left lane), WT OPN1MW-HA (middle lane), or mutant OPN1MW^{C198R} protein (right lane), showing that the antibody against L/M-opsin recognizes both WT and mutant OPN1MW^{C198R} (OPN1MW). TUBA4A was used as a loading control.

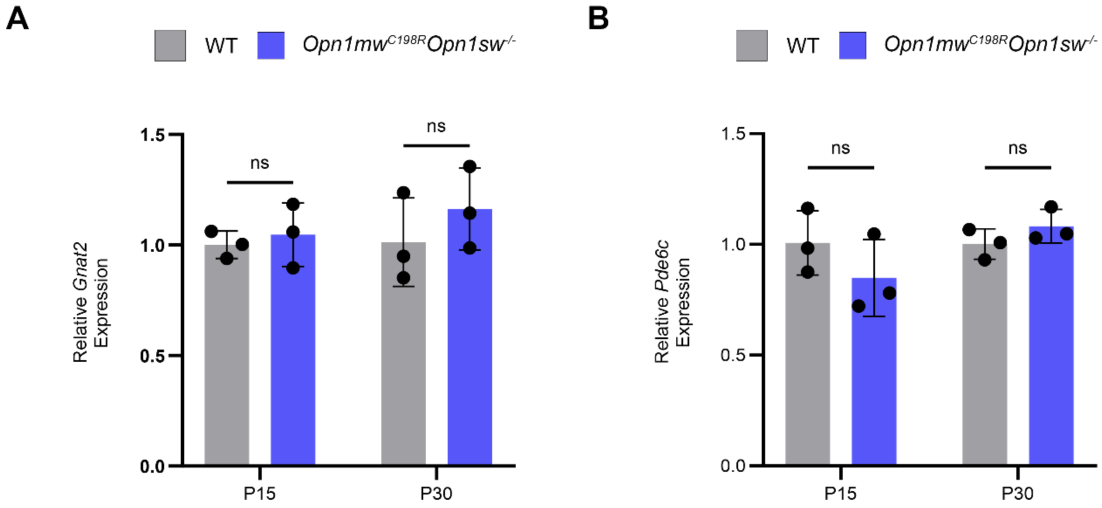

Supplemental Figure 6. Gene expression of *Pde6c* and *Gnat2* is comparable between *Opn1mw*^{C198R}*Opn1sw*$^{-/-}$ and WT at P15 and P30. Real-time qPCR of **(A)** *Pde6c* and **(B)** *Gnat2* mRNA levels in *Opn1mw*^{C198R}*Opn1sw*$^{-/-}$ retinas at P15 and P30 relative to age-matched WT controls. Data represented as mean ± SD, 2-way ANOVA (n = 3; *$p \leq 0.05$, **$p < 0.002$, ***$p < 0.001$).

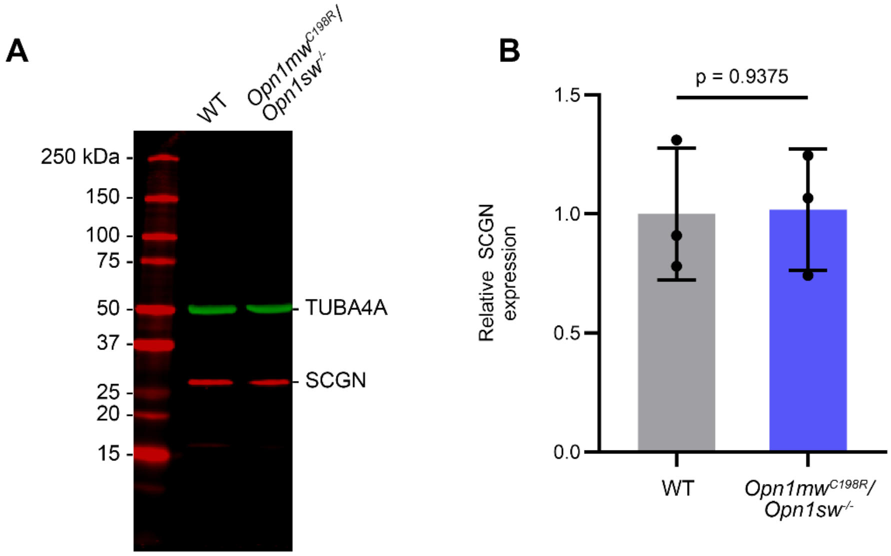

Supplemental Figure 7. ***Opn1mw****^{C198R}****Opn1sw****$^{-/-}$* **cones exhibit normal protein levels of the cone bipolar cell marker secretagogin compared to age-matched WT controls. (A)** Western blot and **(B)** quantification of 10-month old WT (left lane) and *Opn1mw*^{C198R}*Opn1sw*$^{-/-}$ (right lane) retinal lysates with an antibody labeling the cone bipolar cell marker secretagogin (SCGN, red). TUBA4A (green) was used as a loading control. Data represented as mean ± SD, unpaired 2-tailed t-test with Welch's correction (n=3; $p = 0.9375$).
